# Cardiac Tamponade Under Positive-Pressure Ventilation: Pathophysiological Insights and Implications for Diagnosis

**DOI:** 10.31083/RCM49283

**Published:** 2026-06-17

**Authors:** Sara Santangelo, Daniele Orso, Matteo Segat, Alessandro Brussa, Giacomo Bacchetti, Matteo Fabris, Greta Minello, Giorgio Della Rocca

**Affiliations:** ^1^Department of Medicine (DMED), University of Udine, 33100 Udine, Italy; ^2^Department of Emergency, Azienda Sanitaria Universitaria Friuli Centrale (ASUFC), University Hospital “Santa Maria della Misericordia”, 33100 Udine, Italy

**Keywords:** cardiac tamponade, positive-pressure ventilation, transesophageal echocardiography, ventricular interdependence

## Abstract

In mechanically ventilated patients, the clinical and echocardiographic presentation of pericardial tamponade differs from the classic spontaneous-breathing paradigm. Positive-pressure ventilation (PPV) increases pleural and pericardial pressures, narrows the transmural filling gradient, augments ventricular interdependence, and can precipitate tamponade physiology at smaller effusion volumes. Consequently, the classic Doppler cues, such as large respiratory variation in mitral/tricuspid inflow, are commonly blunted, reversed, or absent, and right-sided chamber collapse can be intermittent or phase-shifted across the ventilatory cycle. Therefore, diagnosis depends on integrating clinical suspicion, hemodynamics, and multiple echocardiographic features, including effusion morphology (often loculated/posterior in the postoperative setting), right-atrial systolic and right-ventricular diastolic collapse (timed to the cardiac cycle), venous congestion surrogates (inferior vena cava plethora and superior vena cava/hepatic venous Doppler findings), and direct evidence of reduced stroke volume. Transthoracic echocardiography (TTE) is fast and, when the window is good, can provide a safe guide to life-saving pericardiocentesis. Thus, TTE is established as a first-line approach in both intra-hospital emergencies and in prehospital settings. However, in the context of PPV, tamponade may be underrecognized because suboptimal acoustic windows, postoperative dressings, emphysema, thoracic trauma, or prone positioning can limit TTE. Across 40 physiological, diagnostic accuracy, and impact studies, early TTE after cardiac surgery showed only modest performance in surgically confirmed tamponade (area under the curve of approximately 0.64 and a positive predictive value of approximately 58%). Meanwhile, about half of the postoperative effusions were predominantly posterior or loculated. In mechanically ventilated intensive care unit cohorts, transesophageal echocardiography (TEE) resolved almost all prespecified clinical questions and changed therapy more often than TTE (97% vs. 38% and 36% vs. 16%, respectively). These findings support a physiology-anchored approach in which TTE remains first-line; meanwhile, TEE should be considered early when clinical suspicion is moderate-to-high, and TTE is nondiagnostic or when posterior/loculated or regional tamponade is suspected during PPV.

## 1. Introduction

**Fig. 1.**
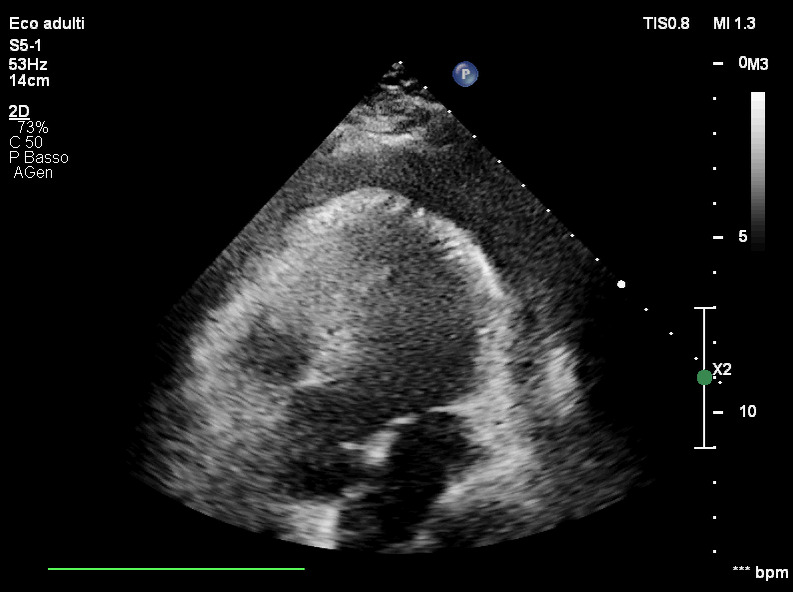
**Transthoracic echocardiography (subcostal five-chamber view) showing a circumferential pericardial effusion**. Representative subcostal TTE image demonstrating an anechoic pericardial fluid layer surrounding the heart, consistent with circumferential pericardial effusion. This first-line view is commonly used in emergency and ICU settings to rapidly confirm effusion presence and guide escalation to hemodynamic assessment and drainage when tamponade physiology is suspected. ICU, intensive care unit; TTE, transthoracic echocardiography; MI, mechanical index; 2D, two-dimensional echocardiography; bpm, beats per minute.

**Fig. 2.**
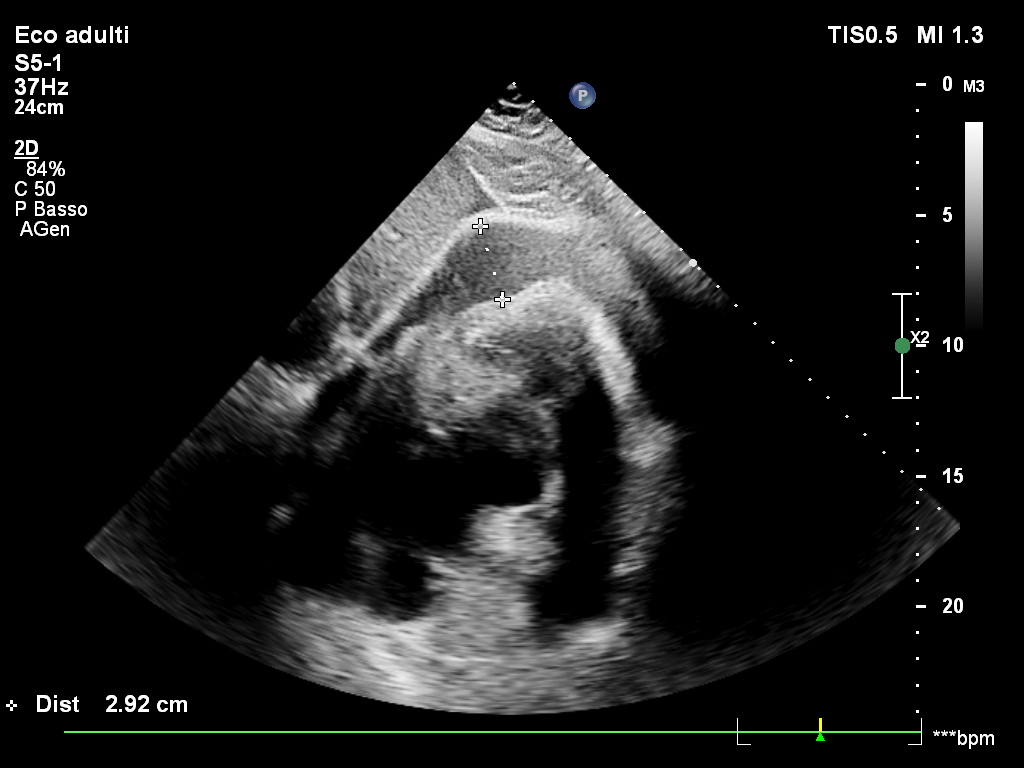
**Transthoracic echocardiography (subcostal view) showing a large circumferential pericardial effusion**. Subcostal TTE image demonstrating a prominent anechoic pericardial fluid layer encircling the heart; caliper measurement indicates an effusion thickness of approximately 2.92 cm. This view is commonly used at the bedside to rapidly quantify effusion size and support urgent evaluation for tamponade physiology in unstable patients.

Cardiac tamponade in patients receiving positive-pressure ventilation (PPV) is a high-stakes emergency in the intensive care unit (ICU), in which cardiopulmonary interactions can mask classical clinical and echocardiographic signs and precipitate abrupt decompensation [1]. Transition to PPV raises pleural and pericardial pressures and can precipitate low-output shock even with modest pericardial effusions, particularly around induction or positive end-expiratory pressure (PEEP) escalation [2,3,4]. In this setting, diagnostic heuristics derived from spontaneously breathing patients—such as marked respiratory variation in mitral and tricuspid inflow and pronounced pulsus paradoxus—are often blunted, reversed, or absent [5]. Recognition of tamponade must therefore rely on integrated interpretation of clinical suspicion, imaging and physiology: inspiratory variations in transmitral and tricuspid flow should be merged with right-atrial systolic and right-ventricular diastolic collapse carefully timed to the cardiac cycle, venous Doppler patterns (superior vena cava [SVC] and hepatic veins), and direct surrogates of stroke volume (e.g., left ventricular outflow tract [LVOT] velocity–time integral) interpreted in the context of hemodynamic compromise [6,7,8,9]. Clinical context and transthoracic echocardiography (TTE) window can guide the choice between percutaneous and surgical drainage, as TTE is fast, requires less technology and expertise and when the window is good can provide a safe guide to life saving pericardiocentesis, as well established in an emergency context in eFAST and in pre-hospital setting (ERC 2025) [10] (Figs. 1,2).

**Fig. 3.**
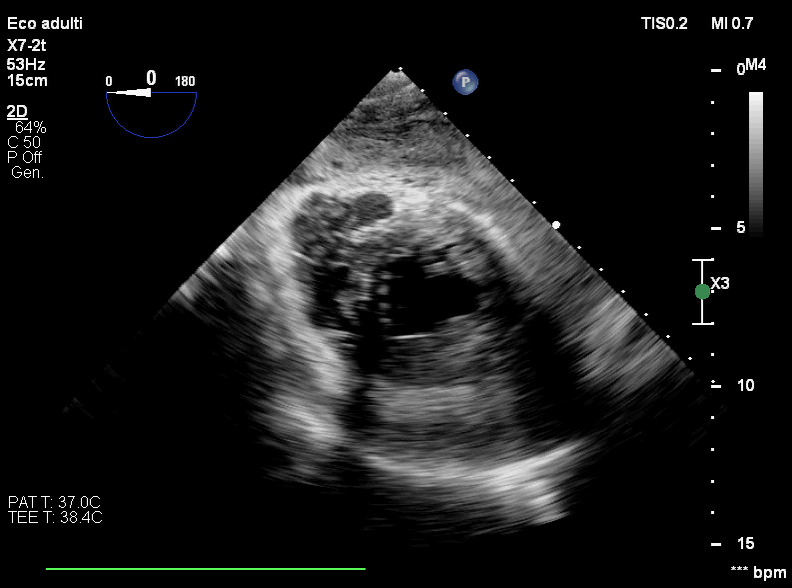
**Transesophageal echocardiography (transgastric short-axis view) showing pericardial effusion with suspected posterior intrapericardial clot**. Transgastric mid–short-axis TEE image demonstrating pericardial fluid with echogenic material along the posterior pericardial space, suggestive of organized clot/loculated postoperative collection. TEE, transesophageal echocardiography.

**Fig. 4.**
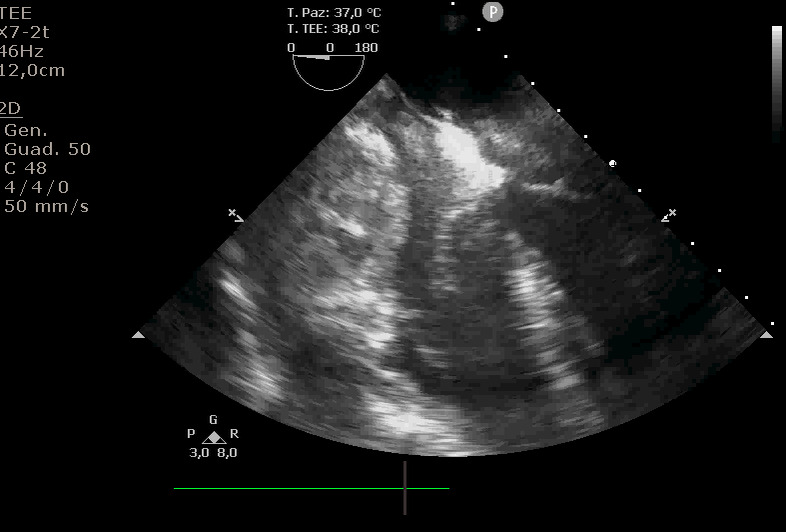
**Transesophageal echocardiography (mid-esophageal four-chamber view) showing pericardial effusion with intrapericardial clot causing right-sided chamber compression**. Mid-esophageal four-chamber TEE image demonstrating pericardial fluid with echogenic organized material consistent with clot, associated with focal external compression of the right atrium and right ventricle—an imaging pattern typical of regional/loculated postoperative tamponade and one that may be underestimated on transthoracic windows.

Post-cardiac surgery anatomy further complicates assessment: posterior clots and loculated effusions frequently produce regional compression that eludes transthoracic echocardiography and is more reliably delineated with transesophageal echocardiography [4,11,12,13] (Figs. 3,4).

Although society guidelines acknowledge these caveats, and many centers use transesophageal echocardiography liberally in ventilated or postoperative patients, clinicians still lack a concise, physiology-anchored framework that explains how PPV reshapes tamponade pathophysiology, recalibrates echocardiographic interpretation, and links imaging with airway and ventilator decisions to shorten time to diagnosis and time to drainage [14,15,16].

Cardiac tamponade in mechanically ventilated patients represents a distinct and clinically high-risk phenotype. PPV can unmask or exacerbate pericardial constraint by reducing venous return and amplifying ventricular interdependence, thereby precipitating abrupt hemodynamic collapse even in cases where classic “textbook” echocardiographic signs are absent or attenuated. In daily critical care and perioperative practice, this scenario is particularly relevant because PPV, sedation, and changes in intrathoracic pressure (e.g., PEEP escalation, recruitment maneuvers, induction of anesthesia, patient–ventilator dyssynchrony) are frequent and may convert a compensated pericardial effusion into a rapidly decompensating state. Importantly, tamponade physiology under PPV may be under-recognized: TTE can be limited by suboptimal acoustic windows, postoperative dressings, emphysema, or prone positioning, and the presence of posterior or loculated effusions—especially after cardiac surgery—may further reduce diagnostic sensitivity. Delayed recognition may lead to persistent low-output shock, inappropriate escalation of fluids/vasopressors/airway pressures, and potentially avoidable peri-intubation or perioperative arrest. For these reasons, a physiology-oriented echocardiographic approach—often requiring transesophageal echocardiography (TEE) and incorporating venous Doppler and stroke-volume surrogates (e.g., LVOT/right ventricular outflow tract (RVOT) velocity–time integral (VTI)) rather than relying exclusively on respiratory variation of atrioventricular inflow—may substantially improve bedside decision-making and time-to-drainage.

Although robust epidemiological estimates are limited by heterogeneity in case definitions, reference standards, and setting-specific risk, several recurring clinical contexts account for a substantial proportion of tamponade under PPV. These include postcardiotomy and post-cardiac surgery patients, in whom effusions are frequently posterior, loculated, or partially organized; mechanically ventilated ICU patients with pre-existing effusions undergoing ventilatory escalation; trauma cohorts with limited acoustic windows; and selected oncologic or postoperative patients with inflammatory or malignant pericardial disease. Across these contexts, underdiagnosis is plausible because PPV can attenuate or invert traditional respiratory Doppler “cut-offs”, regional compression may not produce classic chamber collapse, and TTE may be limited precisely when rapid decisions are needed. A clear, physiology-based approach tailored to PPV is therefore clinically relevant for a broad readership involved in critical care, anesthesia, and perioperative echocardiography.

In this narrative review, we propose a physiology-anchored framework to improve bedside recognition and management of cardiac tamponade in patients receiving PPV. The concepts discussed herein are most applicable to patients receiving invasive PPV in whom pericardial constraint is suspected or confirmed, particularly: (1) postcardiotomy or post–cardiac surgery ICU patients (difficulties in obtaining TTE windows, posterior/loculated effusions, clot/organized collections); (2) ventilated ICU patients with new or worsening shock temporally related to changes in airway pressures (e.g., PEEP escalation, recruitment maneuvers, dyssynchrony); (3) blunt or penetrating chest trauma with suboptimal TTE windows; and (4) oncologic or postoperative patients with pericardial effusion in whom “modest” effusion size may still become hemodynamically significant under PPV. The intended audience includes intensivists, anesthesiologists, cardiologists, and echocardiographers who perform bedside TTE/TEE in acute hemodynamic instability.

### Clinical Vignette

**Fig. 5.**
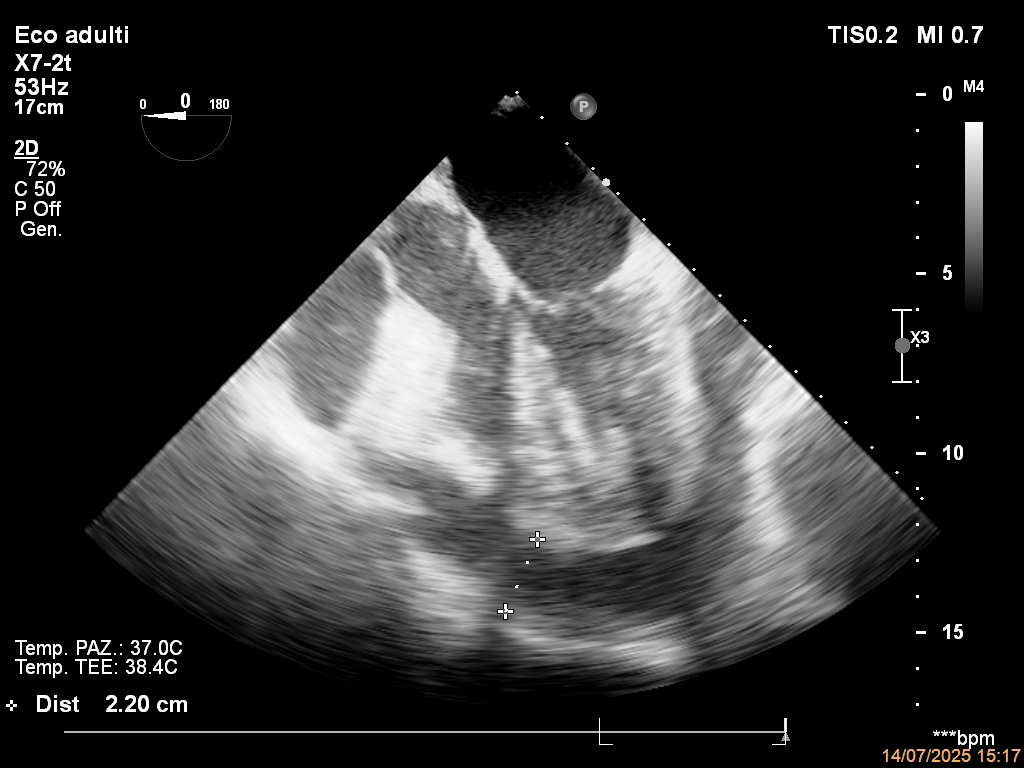
**Pericardial effusion on TEE in a mechanically ventilated patient**. Mid-esophageal four-chamber TEE view demonstrating a pericardial effusion with predominant distribution around the apical/peri-ventricular region (maximal separation ≈22 mm). This example illustrates how clinically relevant effusions may be incompletely characterized on TTE in ventilated/postoperative patients and supports early TEE when TTE is nondiagnostic or when regional/loculated disease is suspected. LV, left ventricle; PPV, positive-pressure ventilation .

**Fig. 6.**
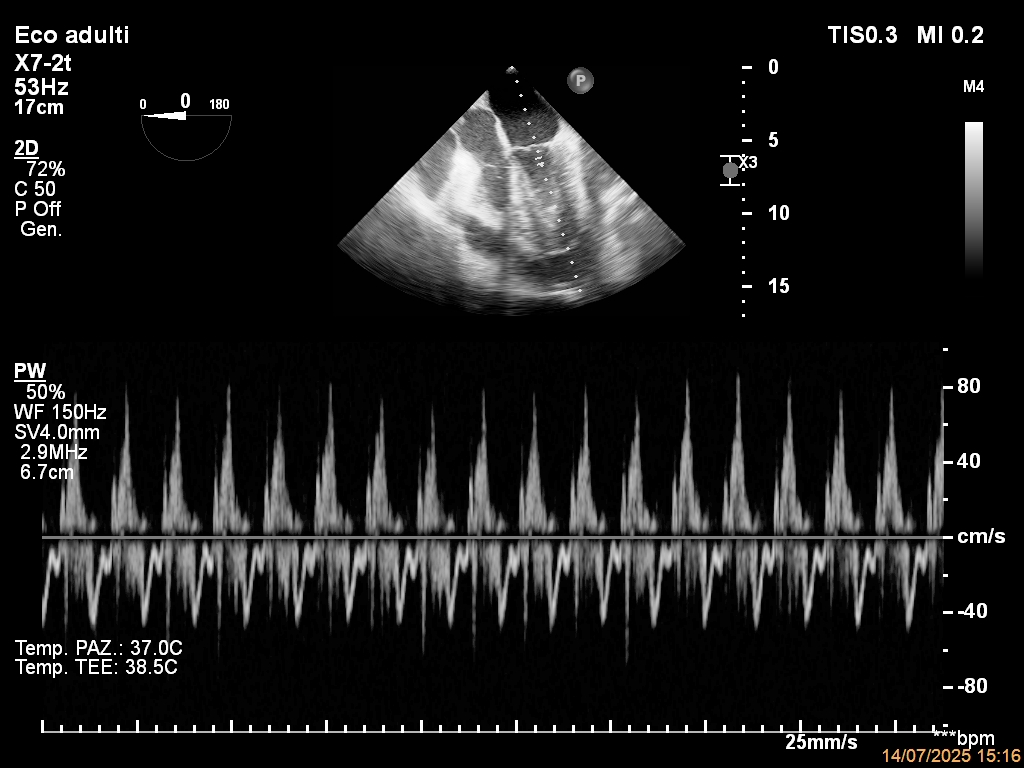
**Blunted transmitral respiratory variation under positive-pressure ventilation**. Transesophageal pulsed‐wave Doppler of mitral inflow in a mechanically ventilated ICU patient with pericardial tamponade, showing fused E/A waves and minimal beat-to-beat (respiratory) variation in peak diastolic velocity under positive-pressure ventilation. Classic respirophasic transmitral cut-offs are therefore not met despite hemodynamically significant tamponade, illustrating the limited reliability of Doppler “pulsus paradoxus” criteria in patients receiving PPV. Abbreviations: E/A, early (E) and late atrial (A) diastolic inflow waves; ICU, intensive care unit.

**Fig. 7.**
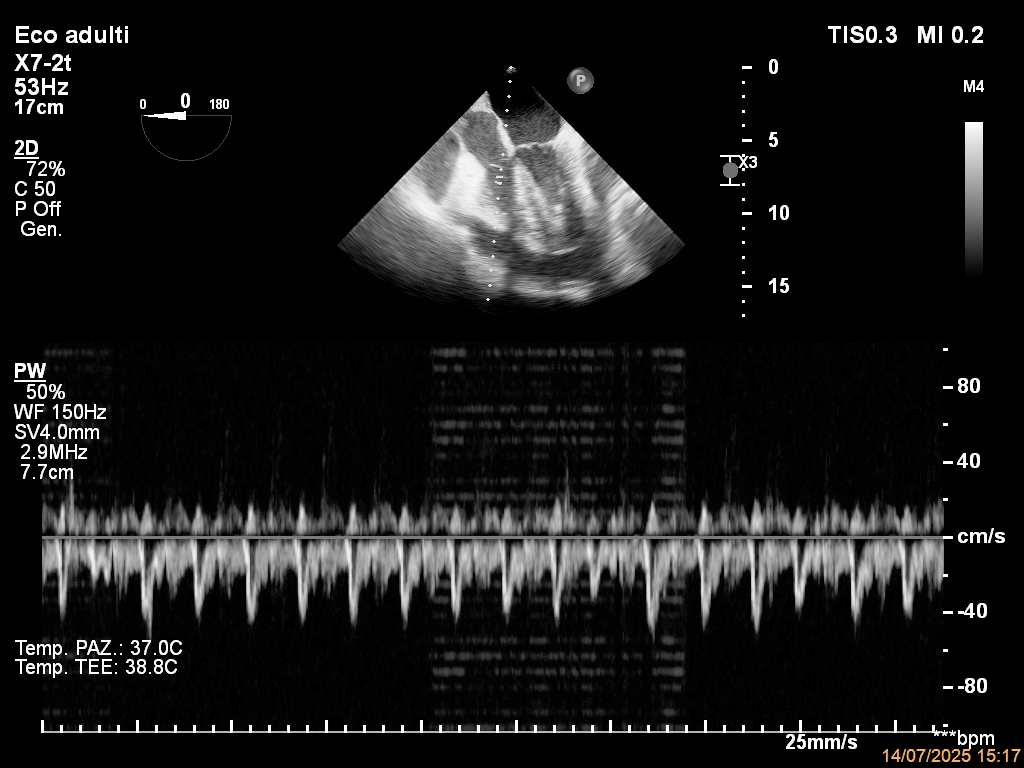
**Blunted tricuspid respiratory variation under positive-pressure ventilation**. Transesophageal pulsed-wave Doppler of tricuspid inflow in the same mechanically ventilated ICU patient with pericardial tamponade, showing only minimal respiratory variation in diastolic flow velocities, illustrating how positive-pressure ventilation can blunt the classic respirophasic Doppler changes of cardiac tamponade.

A mechanically ventilated postcardiotomy ICU patient developed progressive hypotension and rising vasopressor requirements shortly after escalation of PEEP for worsening oxygenation. Bedside transthoracic echocardiography was technically limited by surgical dressings, subcutaneous emphysema, hyperinflated lungs, and suboptimal acoustic windows, and did not demonstrate unequivocal chamber collapse; the effusion size and distribution could not be confidently characterized. Given the temporal relationship between airway-pressure escalation and shock, transesophageal echocardiography was performed and confirmed pericardial effusion with features consistent with clinically relevant pericardial constraint under PPV (Fig. 5). Doppler findings were interpreted in the context of ventilator-phase dependence and the known blunting of traditional respiratory inflow “cut-offs” during PPV (Figs. 6,7). Following prompt surgical drainage, hemodynamics improved with reduced vasopressor requirement. This vignette illustrates why tamponade physiology under PPV may be underestimated on TTE and supports early escalation to TEE when suspicion remains moderate-to-high and TTE is nondiagnostic, particularly after cardiac surgery.

## 2. Literature Review

### 2.1 Search Strategy and Selection Criteria

We searched MEDLINE (Ovid), Embase, Web of Science, Cochrane Library, and Scopus from Jan 1, 1985, to Oct 29, 2025, without language restrictions. Search concepts combined: pericardial effusion; cardiac tamponade; mechanical ventilation/PPV/PEEP; TTE/TEE; postoperative/post-cardiac surgery; regional tamponade; diagnostic accuracy/impact; cardiopulmonary physiology (transmural pressure, venous return, ventricular interdependence, septal mechanics). Inclusion: perioperative/ICU cohorts; diagnostic-accuracy or diagnostic-impact studies; mechanistic/physiological experiments under PPV; major society guidance. Exclusion: single case reports unless mechanistically clarifying; non-data opinion pieces. Reference lists were hand-searched. The full database-specific search strings (controlled vocabulary and free-text terms) are reported in **Supplementary Materials**.

### 2.2 Narrative Review

We structured the narrative synthesis around four prespecified aims: (a) to describe how PPV alters pericardial and transmural pressures, venous return, ventricular interdependence, and septal mechanics [2]; (b) to summarize how these changes modify echocardiographic signs of tamponade (timing of chamber collapse, Doppler behavior, venous surrogates) and what to prioritize under PPV [1]; (c) to appraise the relative contribution of transthoracic and transesophageal echocardiography in ventilated and postoperative patients [17]; and (d) to outline a pragmatic ICU pathway linking physiology, imaging, and drainage.

Study identification is detailed in the Search strategy section; we focused on ICU and perioperative cohorts with mechanically ventilated patients, comparative TTE–TEE data, and mechanistic experiments under PPV. We prioritized contemporary guidance (e.g., American Society of Echocardiography, American Society of Echocardiography/Society of Cardiovascular Anesthesiologists/Society of Thoracic Surgeons, American Heart Association), perioperative Doppler models under PPV, and postcardiotomy series with surgical confirmation; physiology reviews were used to frame mechanisms and terminology rather than as primary evidence for diagnostic performance or outcomes and we present conflicting findings in their clinical context (timing after surgery, effusion morphology) [8]. Reasoning proceeds from first principles (external constraint and venous return) to test performance (echo under PPV) and then to management decisions (TEE-first triggers, indications for drainage). Where available, we report quantitative indices of diagnostic performance and impact on decision-making, grouped by clinical context (ICU ventilated vs postcardiotomy), modality (TTE vs TEE), and reference standard.

## 3. Results

We identified 41 relevant publications spanning 1960–2025, encompassing 20 narrative reviews, guidelines, or position papers (48.7%), 15 original physiological or observational studies (36.5%), and 6 case reports or small case series (14.6%) [1,2,3,4,5,6,7,8,9,10,11,12,13,14,15,16,17,18,19,20,21,22,23,24,25,26,27,28,29,30,31,32,33,34,35,36,37,38,39,40,41]. The largest thematic cluster (15/41; 367.5%) addressed pericardial disease and cardiac tamponade across the perioperative, chronic, and low-pressure settings, including contemporary series of postoperative tamponade and classical descriptions of low-pressure tamponade integrating echocardiographic and hemodynamic profiles [3,4,6,8,9,14,15,16,20,29,33,34,35,37,38]. A second group (9/41; 21.9%) of studies focused on heart–lung interactions during PPV, characterizing the effects of lung inflation, PEEP, and changes in pleural and pericardial pressure on venous return, right ventricular afterload, and transmitral flow, with several intraoperative human studies providing direct measurements of transmural filling pressures and pericardial constraint [1,2,18,19,25,27,30,31,32]. A third cluster (4/41; 9.8%) concentrated on ventricular interdependence and the role of the pericardium as a shared constraint, linking right ventricular filling to left ventricular performance [21,22,26,28]. In parallel, multiple reviews, diagnostic studies, and case reports evaluated transthoracic and transesophageal echocardiography in shock, in mechanically ventilated ICU patients, and after cardiac surgery, including cases of loculated or atrial tamponade missed by standard windows (8/41; 19.5%) [7,11,12,13,17,36,39,40]. Recent conceptual and methodological papers addressed hemodynamic assessment at the bedside—through inferior vena cava (IVC) ultrasonography, velocity–time integral, and venous return/mean systemic filling pressure frameworks—and multimodality imaging recommendations for pericardial disease, providing a coherent physiological scaffold that directly informs our interpretation of tamponade and preload-sensitive indices in ventilated patients (5/41; 12.2%) [5,23,24,41,42].

The available diagnostic-accuracy and impact studies indicate that within the first 24 h after cardiac surgery, transthoracic echocardiography predicts surgically confirmed tamponade with only modest discrimination (area under the curve about 0.64 and a positive predictive value around 58%). Anatomical series report that roughly 50% of effusions are predominantly posterior; therefore, in a prospective ICU cohort of mechanically ventilated patients, transesophageal echocardiography resolved 97% of prespecified clinical questions versus 38% for transthoracic echocardiography and prompted therapeutic changes more often (36% vs 16%). These signals strengthen our emphasis on a physiology-anchored interpretation of echocardiography and a low threshold for early TEE in ventilated or postcardiotomy patients with suspected tamponade when TTE is not conclusive (Table 1).

**Table 1.** **Hemodynamic and echocardiographic differences between cardiac tamponade in spontaneous breathing and during positive-pressure ventilation**.

Feature	Spontaneous breathing	Positive-pressure ventilation
Hemodynamics	Gradual rise in pericardial pressure; larger effusion needed	Higher intrathoracic/pericardial pressure; tamponade with smaller effusion
Pulsus paradoxus	Typical, pronounced	Often blunted or absent
Doppler inflow	Marked respirophasic mitral/tricuspid variation	Variation blunted or inverted; classic cut-offs unreliable
Chamber/venous signs	RA/RV collapse + plethoric IVC usually diagnostic	RA/RV collapse timing altered; IVC nonspecific, systemic venous Doppler more informative
Imaging strategy	TTE often sufficient	Low threshold for first-line use of TEE

Key pathophysiological and echocardiographic differences between cardiac tamponade in spontaneously breathing patients and in those receiving positive-pressure ventilation, highlighting blunting of classic respirophasic signs and the greater reliance on systemic venous Doppler and TEE under PPV. Abbreviations: IVC, inferior vena cava; PPV, positive-pressure ventilation; RA, right atrium; RV, right ventricle; TEE, transesophageal echocardiography; TTE, transthoracic echocardiography.

## 4. Discussion

### 4.1 Core Pathophysiology Under Positive-Pressure Ventilation

**Fig. 8.**
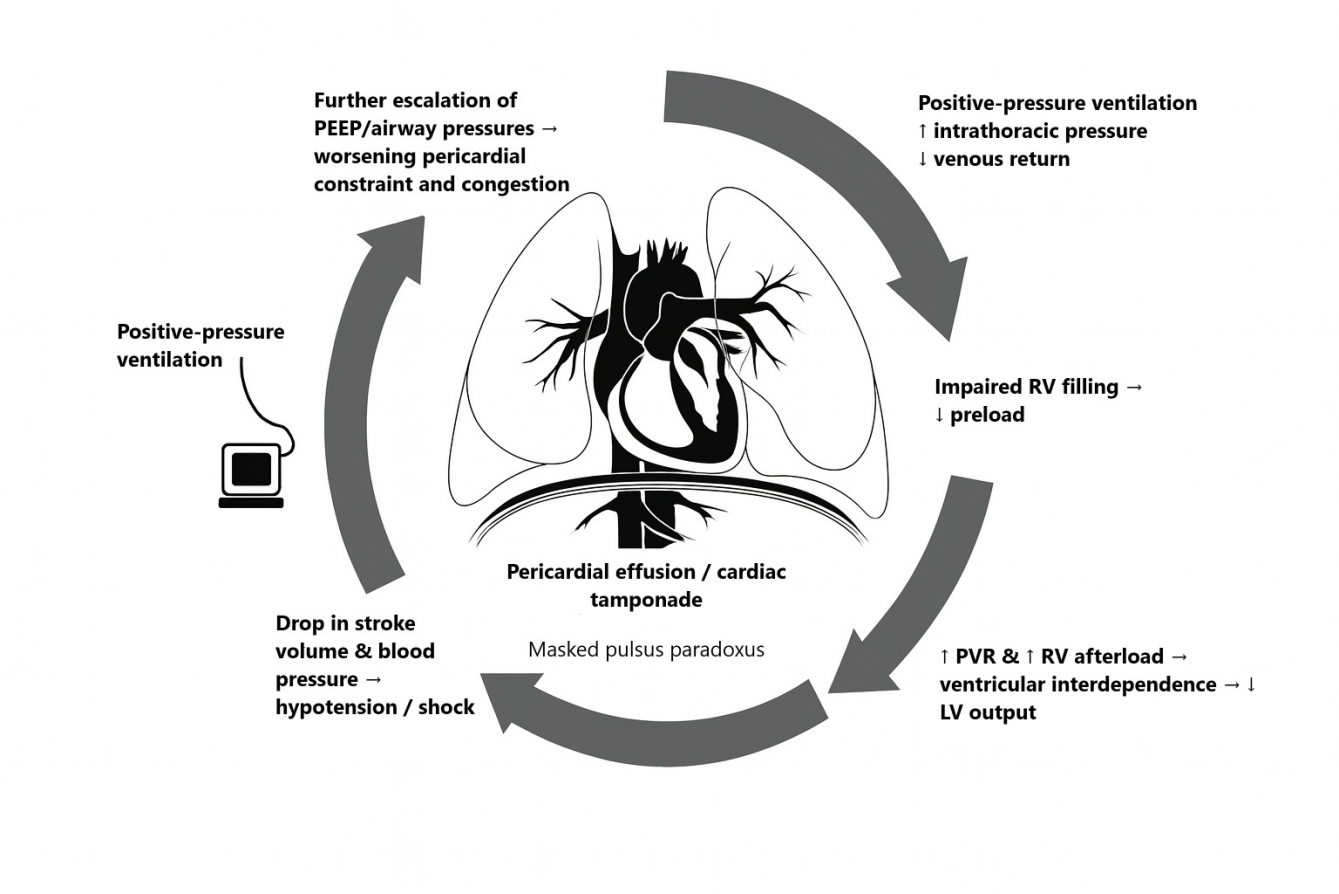
**Pathophysiological vicious cycle of cardiac tamponade under positive-pressure ventilation**. PPV increases intrathoracic pressure and reduces venous return, leading to impaired RV filling, increased pulmonary vascular resistance and RV afterload, ventricular interdependence with reduced LV output, and a fall in stroke volume and blood pressure culminating in hypotension or shock. The circular arrows indicate the self-reinforcing sequence by which PPV and subsequent escalation of airway pressures may progressively amplify hemodynamic compromise in the setting of pericardial constraint. Further escalation of PEEP/airway pressures and fluid loading worsens pericardial constraint and congestion, producing pericardial effusion/cardiac tamponade with masked pulsus paradoxus. Abbreviations: LV, left ventricle; PEEP, positive end-expiratory pressure.

PPV raises pleural pressure, much of which is transmitted to the pericardial space, increasing pericardial pressure (Pperi) and reducing the transmural filling pressure (Ptm ≈ intracavitary pressure − Pperi) [18]. As Pperi approaches right-sided diastolic pressures, diastolic filling becomes exquisitely preload-dependent [19]. The pericardium’s nonlinear pressure–volume relation explains the classic observation that draining a small volume from a tense sac produces a large fall in Pperi and immediate hemodynamic recovery [20] (Fig. 8). Human intraoperative measurements and experimental work demonstrate that pericardial pressure tracks right-atrial pressure and shifts ventricular diastolic mechanics upward; the same load transmitted by PPV can therefore precipitate tamponade at smaller effusion volumes than in spontaneous breathing [21,22].

Echo consequences in patients receiving PPV include right-atrial systolic and right-ventricular diastolic collapse that still occur but can be intermittent or phase-shifted (often most apparent near end-expiration), and their extent can be attenuated when right-sided filling pressures are elevated. Precise timing of these events to the cardiac cycle—such as aligning M-mode with atrioventricular valve motion—remains essential for diagnosing tamponade under these conditions [6,9].

### 4.2 Venous Return Under Positive-Pressure Ventilation: the Guyton Framework in the ICU

Since venous return (VR) is generated by the pressure gradient between mean systemic filling pressure (Pmsf) and right atrial pressure (RAP) over the resistance to venous return (Rvr), PPV and PEEP—which raise pleural pressure and increase RAP—narrow this venous return gradient; auto-PEEP and dynamic hyperinflation can further exacerbate this effect [23]. In hypovolemia (low stressed volume and low Pmsf), the gradient collapses; in tamponade, RAP rises toward Pperi, compounding the fall in VR and reducing stroke volume [24]. Bedside inspiratory/expiratory holds can estimate Pmsf and illustrate the venous return curve, which helps explain why PPV may be poorly tolerated as pericardial constraint increases [25]. An illustrative TEE example of pericardial effusion in a mechanically ventilated patient is shown in Fig. 5.

Under PPV and PEEP, a plethoric IVC mainly reflects raised RAP and becomes a nonspecific sign. Systemic venous Doppler (hepatic, portal, or renal venous flow) likewise reflects the hemodynamic consequences of elevated RAP and reduced right-sided compliance and is not, in isolation, etiologic. Its practical value in ventilated patients is as an additional, quantifiable marker of impaired right-sided filling when integrated with evidence of pericardial constraint (including regional/loculated collections), ventricular interdependence, and ventilator-phase dependence, and when tracked dynamically during airway-pressure adjustments and after pericardial drainage [5,9].

### 4.3 Hepatic Venous Flow Assessment

The normal hepatic venous flow pattern shows a biphasic wave followed by a reversal wave corresponding to atrial contraction.

Burstow et al. [43] showed that in cardiac tamponade the characteristic venous flow pattern consists of a marked predominance of the systolic component, a reduced increase in flow during inspiration, and inversion of the diastolic component during the first expiratory cardiac cycle.

In a study by Mercé and colleagues [44], abnormal venous flow showed a good correlation with the clinical features of cardiac tamponade, with higher sensitivity than right ventricular collapse and much higher specificity than right atrial collapse. The specificity further increases when associated with right chamber collapse [44].

Positive-pressure ventilation alters respiratory variations in hepatic venous flow as well; therefore, it reduces the reliability of Doppler signs based on respiratory changes. However, some features may retain diagnostic value, such as inversion of the hepatic diastolic flow and a markedly pulsatile pattern associated with venous congestion.

It should be emphasized that with the transesophageal technique the hepatic veins can be visualized in nearly all cases, whereas with transthoracic echocardiography they cannot be adequately assessed in approximately one third of patients [45].

### 4.4 Ventricular Interdependence and the Interventricular Septum

The ventricles share the pericardium and the interventricular septum; changes in volume/pressure on one side alter the other—ventricular interdependence [27]. Under PPV, lung inflation increases RV outflow impedance (pulmonary vascular resistance) and reduces RV stroke volume; the trans-septal pressure gradient (left ventricular pressure – right ventricular pressure) oscillates, and the septum shifts accordingly. In tamponade, pericardial constraint accentuates these shifts: small inspiratory changes in RV pressures cause excessive septal excursion (the ‘septal bounce’ or inspiratory bulge into the LV), reducing LV diastolic compliance and magnifying respirophasic swings [27,28].

Under PPV, the phase of septal motion can invert compared with spontaneous breathing, and its magnitude can be attenuated when intrathoracic and pericardial pressures predominate. Septal kinetics should therefore be interpreted in conjunction with stroke-volume surrogates (e.g., LVOT velocity–time integral) rather than in isolation [27].

### 4.5 Why the Classic Doppler Pulsus Paradoxus Fails (or Flips) Under Positive-Pressure Ventilation

In spontaneous-breathing tamponade, inspiration increases RV filling and shifts the septum toward the LV, decreasing transmitral inflow and increasing tricuspid inflow [29]. Under PPV, inspiratory positive pleural pressure reduces systemic venous return and increases RV afterload, while impelling pulmonary venous blood toward the LA; patterns may invert, and as pericardial constraint intensifies, respiratory variation in transmitral flow becomes minimal or absent. A perioperative model quantified this: transmitral E-wave variation fell to ~16% in true tamponade during standard PPV—almost absent—explaining why classic Doppler cut-offs are unreliable in ventilated patients [1] (Fig. 6). A similar blunting can be observed on transtricuspid inflow under PPV (Fig. 7). American Society of Echocardiograpy guidance explicitly cautions that minimal mitral inflow variation is expected in PPV-tamponade [8].

### 4.6 The Mechanics of the Chest Wall, Lung Volume, and Right Ventricular Afterload

PPV increases transpulmonary pressure and stretches alveolar vessels, raising pulmonary vascular resistance and RV afterload—especially at high lung volumes or with poor pulmonary compliance [27,30]. Cyclic increases in RV outflow impedance during tidal ventilation have been demonstrated *in vivo* with echo-Doppler [31]. Chest-wall compliance modulates how much airway pressure becomes pleural pressure; with a stiff chest wall (e.g., abdominal hypertension), a larger fraction of airway pressure transmits to pleura and pericardium, tightening the external constraint on the heart [32]. These interactions explain the hemodynamic fragility of tamponade during PPV and the heterogeneity of response to PEEP.

**Fig. 9.**
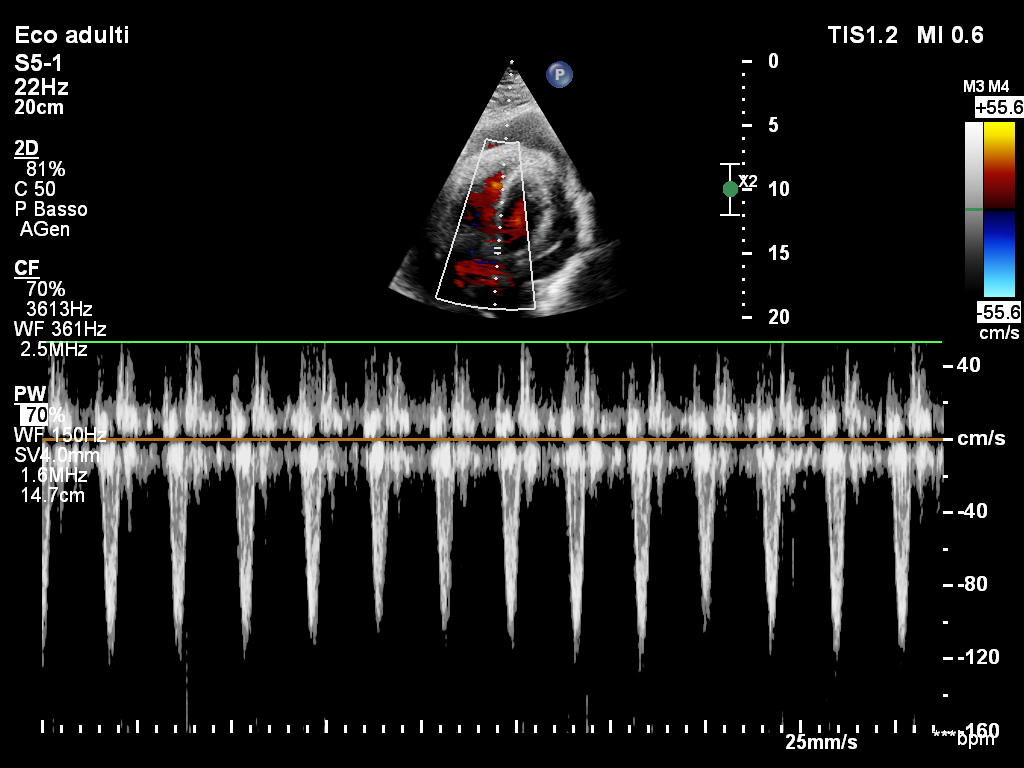
**LVOT VTI under positive-pressure ventilation in cardiac tamponade physiology**. Pulsed-wave Doppler of the LVOT shows marked respiratory variation in systolic ejection, consistent with exaggerated ventricular interdependence and ventilator-phase–dependent hemodynamic fluctuation. Abbreviations: LVOT, left ventricular outflow tract; VTI, velocity–time integral.

Under PPV, cyclic variation in RVOT VTI with phase-linked septal motion is expected; these changes reflect the respiratory swings in right ventricular afterload and filling. LVOT VTI (or, where appropriate, RVOT VTI) can be followed as a quantitative surrogate of stroke volume when titrating PEEP or driving pressure and after pericardial drainage [31] (Fig. 9).

### 4.7 Low-Pressure Tamponade and the ‘Masked’ Clinical Picture Under Positive-Pressure Ventilation

When intravascular volume is low (low Pmsf) and PPV raises RAP/Pperi, even modest effusions can equilibrate with chamber pressures (low-pressure tamponade). Under sedation/PPV, classic bedside signs (pulsus paradoxus, jugular vein distension) may be attenuated or misleading, pushing the diagnostic burden onto physiological-congruent echocardiography (collapse timing, regional compression, venous Doppler) and output metrics [33,34]. Contemporary critical-care guidance warns that PPV is poorly tolerated and may unmask/worsen tamponade [8,35].

### 4.8 Regional Tamponade Anatomy Explains Why TEE is Often Necessary

After cardiac surgery, posterior loculations or clot can selectively compress the left atrium, posterior LV, or pulmonary veins—often with minimal global signs on TTE. Accuracy for surgically confirmed tamponade is modest in the first 24 h (area under the curve ~0.64; positive predictive value ~58%) and improves later [36]; anatomical series show about 50% posterior effusions—precisely the pathology TTE tends to miss, and TEE depicts [4,37,38].

In intubated or postcardiotomy patients, TTE windows are frequently poor, and pathology is often posterior/loculated. Authoritative recommendations endorse a low threshold for TEE when TTE is limited or regional compression is suspected, and they caution against reliance on respiratory inflow variation under PPV. In a prospective ICU cohort of mechanically ventilated patients, TEE resolved 97% of prespecified clinical questions versus 38% for TTE and changed therapy more often (36% vs 16%), including prompting urgent surgery [39,40].

### 4.9 Hemodynamic Echocardiography in Tamponade Under Positive-Pressure Ventilation

In a patient with a known pericardial effusion, new hypotension or a fall in LVOT VTI temporally linked to induction or increases in PEEP/driving pressure should increase the pretest probability of tamponade rather than be ascribed simply to “ventilator” or “anesthetic” effects [41,42]. Echocardiography should then be used to explain the hemodynamics rather than to confirm a single sign: confirm the effusion, time right-atrial systolic and right-ventricular diastolic collapse against the cardiac cycle, document septal motion in relation to the respiratory phase, interrogate systemic venous Doppler (SVC/hepatic), and quantify stroke volume with LVOT VTI before and after key interventions [1,9,16]. When transthoracic windows are poor, the patient is ventilated, or postcardiotomy anatomy suggests posterior or loculated disease, TEE should be used early to map posterior recesses, identify regional compression, and sample SVC/hepatic flow; once effusion, chamber compression, and low output coexist, drainage should be rapidly provided (percutaneous or surgical according to anatomy), airway pressures minimized while temporizing, and imaging repeated to document resolution of collapse, a rise in LVOT VTI, and the absence of residual loculations [8,39].

## 5. Limitations

The evidence base spans diagnostic-accuracy, diagnostic-impact, and mechanistic studies with heterogeneous reference standards (surgical confirmation vs clinical adjudication), inconsistent reporting of ventilator settings (especially PEEP and driving pressure), and time-dependent postoperative anatomy (evolving clot and loculation). These features preclude formal meta-analysis and limit the precision of pooled estimates; we therefore report quantitative indices only where they are methodologically robust and place greater weight on internally coherent physiological reasoning.

Potential selection and citation bias, as well as author interpretation bias, were mitigated by an explicit search strategy, anchoring key inferences to contemporary guidelines, and a balanced presentation of discordant findings — for example, the reduced discriminative performance of echocardiography within the first 24 h after cardiac surgery.

## 6. Conclusions

Cardiac tamponade under PPV is not simply “standard” tamponade in a different setting: pleural and pericardial pressures, ventricular interdependence, and postoperative anatomy blunt classic signs and erode the reliability of respiratory Doppler cut-offs. In ventilated and postcardiotomy patients, diagnosis should rest on concordant physiology and imaging — effusion morphology, chamber compression, venous Doppler, and stroke-volume surrogates such as LVOT VTI — with a deliberately low threshold for early TEE when windows are poor or regional disease is likely, and prompt drainage once low output and compression coexist.

Priorities for future research include prospective PPV-stratified accuracy studies of right atrium/right ventricular collapse, venous Doppler, and LVOT VTI with uniform reference standards; comparative pathways testing TEE-first versus TTE-then-TEE in ventilated and post-cardiac-surgery populations, powered for time-to-diagnosis, time-to-drainage, and outcomes; and mechanistic work linking transmural pressure and septal kinetics across graded PEEP to practical, ventilator-specific echocardiographic thresholds.

## Abbreviations

E/A, early and late atrial diastolic inflow waves; eFAST, extended Focused Assessment with Sonography in Trauma; ERC, European Resuscitation Council; ICU, intensive care unit; IVC, inferior vena cava; LA, left atrium; LV, left ventricle; LVOT, left ventricular outflow tract; PEEP, positive end-expiratory pressure; Pmsf, mean systemic filling pressure; PPV, positive-pressure ventilation; Pperi, pericardial pressure; Ptm, transmural pressure; RA, right atrium; RAP, right atrial pressure; Rvr, resistance to venous return; RV, right ventricle; RVOT, right ventricular outflow tract; SVC, superior vena cava; TEE, transesophageal echocardiography; TTE, transthoracic echocardiography; VR, venous return; VTI, velocity–time integral.
