# Supplementary material for: Cardiac Tamponade Under Positive-Pressure Ventilation: Pathophysiological Insights and Implications for Diagnosis
Source: Rev Cardiovasc Med. 2026 Jun 17;27(6):49283. doi: 10.31083/RCM49283 (PMC13339243; doi:10.31083/RCM49283)
Supplement: Supplementary file 1 [file 2153-8174-27-6-49283-s1.zip › Supplementary Material.docx]

**Supplementary Materials. Full search strategy (database-specific queries)**

**Overview**

Searches were conducted in **MEDLINE (Ovid)**, **Embase (Ovid)**, **Web of Science Core Collection**, **Cochrane Library**, and **Scopus** from **1 January 1985 to 29 October 2025**. No language restrictions were applied. The strategy combined controlled vocabulary (MeSH/Emtree where applicable) and free-text terms for the following concepts:

1. **Condition:** pericardial effusion / cardiac tamponade / pericardial disease
2. **Exposure/Context:** positive-pressure ventilation / mechanical ventilation / PEEP / airway pressure / intrathoracic pressure
3. **Index tests / modality:** echocardiography / transthoracic / transesophageal / Doppler (optional)
4. **Settings (optional):** ICU / perioperative / postcardiotomy / postoperative / cardiac surgery / trauma (used as sensitivity narrowing only if needed)

The final queries were adapted to each database’s indexing and syntax.

- **MEDLINE (Ovid) — search string**

**Database/platform:** MEDLINE (Ovid)
**Time limits:** 1985–2025 (01 Jan 1985 to 29 Oct 2025)
**Language limits:** none

1. exp Cardiac Tamponade/

2. exp Pericardial Effusion/

3. exp Pericarditis/

4. (tamponad* or "cardiac tamponad*" or "pericardial tamponad*" or "pericardial effusion*" or hemopericard* or "pericardial constraint" or "regional tamponad*" or loculat*).ti,ab,kw.

5. 1 or 2 or 3 or 4

6. exp Respiration, Artificial/

7. exp Positive-Pressure Respiration/

8. exp Ventilation, Mechanical/

9. (mechanical ventilation or ventilat* or "positive pressure ventilation" or PPV or PEEP or "positive end-expiratory pressure" or "airway pressure*" or "intrathoracic pressure").ti,ab,kw.

10. 6 or 7 or 8 or 9

11. exp Echocardiography/

12. exp Echocardiography, Transesophageal/

13. exp Echocardiography, Doppler/

14. (echocardiograph* or ultrasound or TTE or transthoracic or TEE or transesophageal or doppler).ti,ab,kw.

15. 11 or 12 or 13 or 14

16. (postoperative or postcardiotomy or "cardiac surgery" or CABG or sternotom* or ICU or "intensive care" or perioperat* or trauma).ti,ab,kw.

17. ("diagnostic accuracy" or "diagnostic impact" or sensitivity or specificity or ROC or AUC).ti,ab,kw.

18. ("transmural pressure" or venous return or "ventricular interdependence" or septal mechanic*).ti,ab,kw.

19. 5 and 10 and 15

20. 19 and (16 or 17 or 18) [broadening block reflecting prespecified aims]

21. limit 20 to yr="1985 - 2025"

**- Embase — search string**

**Database/platform:** Embase (as searched)

**Time limits:** 1985–2025

**Language limits:** none

1. exp cardiac tamponade/

2. exp pericardial effusion/

3. exp pericarditis/

4. (tamponad* or "cardiac tamponad*" or "pericardial tamponad*" or "pericardial effusion*" or hemopericard* or "pericardial constraint" or "regional tamponad*" or loculat*).ti,ab,kw.

5. 1 or 2 or 3 or 4

6. exp artificial ventilation/

7. exp positive pressure ventilation/

8. exp positive end expiratory pressure/

9. (mechanical ventilation or ventilat* or "positive pressure ventilation" or PPV or PEEP or "positive end-expiratory pressure" or "airway pressure*" or "intrathoracic pressure").ti,ab,kw.

10. 6 or 7 or 8 or 9

11. exp echocardiography/

12. exp transesophageal echocardiography/

13. exp doppler echocardiography/

14. (echocardiograph* or ultrasound or TTE or transthoracic or TEE or transesophageal or doppler).ti,ab,kw.

15. 11 or 12 or 13 or 14

16. (postoperative or postcardiotomy or "cardiac surgery" or CABG or sternotom* or ICU or "intensive care" or perioperat* or trauma).ti,ab,kw.

17. ("diagnostic accuracy" or "diagnostic impact" or sensitivity or specificity or ROC or AUC).ti,ab,kw.

18. ("transmural pressure" or venous return or "ventricular interdependence" or septal mechanic*).ti,ab,kw.

19. 5 and 10 and 15

20. 19 and (16 or 17 or 18)

21. limit 20 to yr="1985-2025"

**- Web of Science Core Collection — search string**

**Database/platform:** Web of Science Core Collection
**Timespan:** 1985–2025
**Search field:** Topic (TS)

TS=(

(tamponad* OR "cardiac tamponad*" OR "pericardial tamponad*" OR "pericardial effusion*" OR hemopericard* OR "pericardial constraint" OR "regional tamponad*" OR loculat*)

AND

("positive pressure ventilation" OR "mechanical ventilation" OR ventilat* OR PPV OR PEEP OR "positive end-expiratory pressure" OR "airway pressure*" OR "intrathoracic pressure")

AND

(echocardiograph* OR ultrasound OR TTE OR transthoracic OR TEE OR transesophageal OR doppler)

)

AND TS=(postoperative OR postcardiotomy OR "cardiac surgery" OR ICU OR "intensive care" OR perioperat* OR trauma OR "diagnostic accuracy" OR "diagnostic impact" OR "transmural pressure" OR "venous return" OR "ventricular interdependence" OR septal)

**- Cochrane Library — search string**

**Database/platform:** Cochrane Library (CENTRAL and/or Cochrane Reviews)

**Timespan:** 1985–2025

(tamponad* OR "cardiac tamponad*" OR "pericardial tamponad*" OR "pericardial effusion*" OR hemopericard* OR "pericardial constraint" OR "regional tamponad*" OR loculat*):ti,ab,kw

AND

("positive pressure ventilation" OR "mechanical ventilation" OR ventilat* OR PPV OR PEEP OR "positive end-expiratory pressure" OR "airway pressure*" OR "intrathoracic pressure"):ti,ab,kw

AND

(echocardiograph* OR ultrasound OR TTE OR transthoracic OR TEE OR transesophageal OR doppler):ti,ab,kw

AND

(postoperative OR postcardiotomy OR "cardiac surgery" OR ICU OR "intensive care" OR perioperat* OR trauma OR "diagnostic accuracy" OR "diagnostic impact" OR "transmural pressure" OR "venous return" OR "ventricular interdependence" OR septal):ti,ab,kw

**- Scopus — search string**

**Database/platform:** Scopus

**Timespan:** 1985–2025

**Search field:** TITLE-ABS-KEY

TITLE-ABS-KEY(

(tamponad* OR "cardiac tamponad*" OR "pericardial tamponad*" OR "pericardial effusion*" OR hemopericard* OR "pericardial constraint" OR "regional tamponad*" OR loculat*)

AND

("positive pressure ventilation" OR "mechanical ventilation" OR ventilat* OR PPV OR PEEP OR "positive end-expiratory pressure" OR "airway pressure*" OR "intrathoracic pressure")

AND

(echocardiograph* OR ultrasound OR TTE OR transthoracic OR TEE OR transesophageal OR doppler)

AND

(postoperative OR postcardiotomy OR "cardiac surgery" OR ICU OR "intensive care" OR perioperat* OR trauma OR "diagnostic accuracy" OR "diagnostic impact" OR "transmural pressure" OR "venous return" OR "ventricular interdependence" OR septal)

)

AND (PUBYEAR > 1984 AND PUBYEAR < 2026)
